# Unicompartmental Knee Arthroplasty Enables Near Normal Gait at Higher Speeds, Unlike Total Knee Arthroplasty

**DOI:** 10.1016/j.arth.2013.07.036

**Published:** 2013-10

**Authors:** Anatole V. Wiik, Victoria Manning, Robin K. Strachan, Andrew A. Amis, Justin Peter Cobb

**Affiliations:** a MSk Lab, Imperial College, London, England; b Orthopaedic Department, Imperial College NHS Trust, London, England; c Mechanical Engineering, Imperial College, London, England

**Keywords:** gait, walking speed, stride length, unicompartmental knee arthroplasty, total knee arthroplasty

## Abstract

Top walking speed (TWS) was used to compare UKA with TKA. Two groups of 23 patients, well matched for age, gender, height and weight and radiological severity were recruited based on high functional scores, more than twelve months post UKA or TKA. These were compared with 14 preop patients and 14 normal controls. Their gait was measured at increasing speeds on a treadmill instrumented with force plates. Both arthroplasty groups were significantly faster than the preop OA group. TKA patients walked substantially faster than any previously reported series of knee arthroplasties. UKA patients walked 10% faster than TKA, although not as fast as the normal controls. Stride length was 5% greater and stance time 7% shorter following UKA — both much closer to normal than TKA. Unlike TKA, UKA enables a near normal gait one year after surgery.

Unicompartmental Knee Arthroplasty (UKA) is a cost effective alternative to Total Knee Arthroplasty (TKA) [1,2]. However despite well documented long term functional success [3,4], this perceived functional superiority does not currently offset the paradoxical higher revision rate reported in all registries [5]. Functional scores such as the Oxford Knee Score (OKS) do not allow investigators to distinguish reliably between forms of arthroplasty which might offer clinically significant advantages, owing to ceiling effects in several key variables [6]. Difficulty with walking is one of the cardinal symptoms of osteoarthritis of the knee, so the measurement of this as a continuous variable seems appropriate. It has been used to document the difference between patients following TKA at around 0.9 m/s and normal controls at 1.2 m/s in gait laboratories [7]. We surmised that gait at higher speed might provide a surrogate for global knee function. Our primary hypothesis was that we would detect no difference between the gait of the different types of knee arthroplasty. Our secondary hypothesis was that both these procedures would restore near normal gait.

## Methods

Sixty subjects were tested on an instrumented treadmill, 3 groups were measured (23 with well functioning unilateral UKAs, 23 with well functioning unilateral TKAs and 14 healthy controls). The two high performing arthroplasty groups were recruited based on high OKS, and were well age and gender matched (Table 1). All knee arthroplasty subjects were a minimum of 12 months post-arthroplasty and had been discharged from routine follow up. All were operated upon by the two senior surgical authors, one who performs UKA whenever possible in patients presenting for knee arthroplasty with osteoarthritis, and the other who primarily performs TKA for this condition, only very rarely performing UKA in highly selected cases. After informed consent each subject was tested by an observer, who was blinded to the intervention performed. After a 6 min acclimatization period at 4 km/h on the instrumented treadmill (Kistler Gaitway, Kistler Instrument Corporation, Amherst, NY), the speed was then increased incrementally until either the subject felt uncomfortable, or the gait performance had deteriorated [8]. The procedure generally took 12 min of continuous walking without any safety rail assistance and was completed without difficulty by all subjects. At all incremental intervals of speed, the vertical component of the ground reaction forces, center of pressure and temporal measurements were collected for both limbs with a sampling frequency of 100 Hz over 10 s. Body weight and Hof scaling [9] were also applied to the outputted mechanical data to correct for mass differences and leg length, respectively. All treadmill outputted mechanical data were subject to averaging to handle the large amount of continuous data being exported, as 10 s interval normally entailed a minimum of 6 steps for each limb. All variables for each subject group were compared to each other using a Student-T test and a significance was set at α = 0.05.

## Results

The two experimental groups were matched for age, gender, height and BMI (Table 1); however the Oxford knee scores of the UKA group were higher than the TKA group, in keeping with other findings following these two forms of arthroplasty [4]. At top walking speed (TWS) the UKA group was able to walk significantly faster, achieving a mean of 6.95 km/h (1.93 m/s) compared to the TKA group with a mean of 6.16 km/h (1.71 m/s). Hof scaling confirmed the substantial difference between the two experimental groups (Table 2). The 11% difference detected in speed appeared to be due to the reduced step length, increased stance time and a lower cadence in the TKA group (Table 2). The UKA group appeared to be almost as efficient as the healthy controls with regards to the impulse per step, and ground reaction forces at heel strike (Fig.), while the TKA was significantly less effective in both metrics. However, at both mid stance, when the joint is unloaded, and toe off when a second peak of force is delivered, neither form of arthroplasty matched the normal gait cycle.

## Discussion

This is a small study of patients discharged from routine follow up at a minimum of one year following surgery. It must be repeated that the groups were operated upon by different surgeons in the same institution, but both of those surgeons performed the operations chosen in large numbers. While selection bias remains a major problem, and could explain many of the findings in this study, the standard of surgery performed should not be a factor. Both the UKA and TKA groups had functional scores that were similar to those reported in larger studies following those procedures, confirming our assumption that the operations were performed well, while the normal controls were age and gender matched.

Prior to this study, the gait of patients with knee arthroplasties of different sorts has been documented in a gait laboratory with data suggesting that the presence of a PCL might have some benefit [10], although still significantly different from normal at normal walking speeds [7], with reduced flexion compared and stride length. Unicompartmental knees have also been studied, and reported to be closer to normal [11]. However to our knowledge, a direct comparison of the two forms of arthroplasty has not been carried out.

Our primary hypothesis was that there would be no difference in the gait of people with different forms of arthroplasty. By giving patients time to increase their speed gradually, we hoped to highlight any differences, overturning our null hypothesis. This technique enabled our TKA patients to attain speeds that were twice as fast as those reported from conventional gait labs [7] at 6.2 km/h. The UKA patients were 11% faster at 7.0 km/h, although still slower than the age and gender matched control group at 7.4 km/h. These differences were all significant. Of the individual variables, perhaps of greatest interest is the stride length: the UKA group had almost identical stride lengths, while the stride length of the TKA group was 10% shorter. This suggests that our secondary hypothesis was partly satisfied. In some respects, UKA can restore near normal gait, but not in all. We have not been able to demonstrate that TKA does this.

This is not randomized data, so conclusions must be cautious, but comparing the two arthroplasty groups with very similar demographics, it appears that UKA may have some functional advantage over TKR as suggested by their higher functional scores. We have reported the best function by any TKA in the literature, but using this demanding methodology, we have yet to find a TKA whose gait approximates that of the normal controls, in terms of speed, stride length and cadence while many UKA patients do so. The elements of gait in which TKA appears to differ markedly from normal include Heel Strike, Step Length, Stance Time and Cadence, while UKA appears to match the normal gait more closely in these variables. The simple metric of top walking speed has some merit for comparing outcomes of arthroplasty, as it is a linear variable. Its use may help distinguish between the gaits associated with different forms of total knee arthroplasty, and give insight into the value of different elements of the knee and their contribution towards gait and global function.

Conflict of Interest Statements

HP0006.

Conflict of Interest.

HP0007.

HP0008.

Conflict of interest statement.

COI (2).

## Figures and Tables

**Fig:**
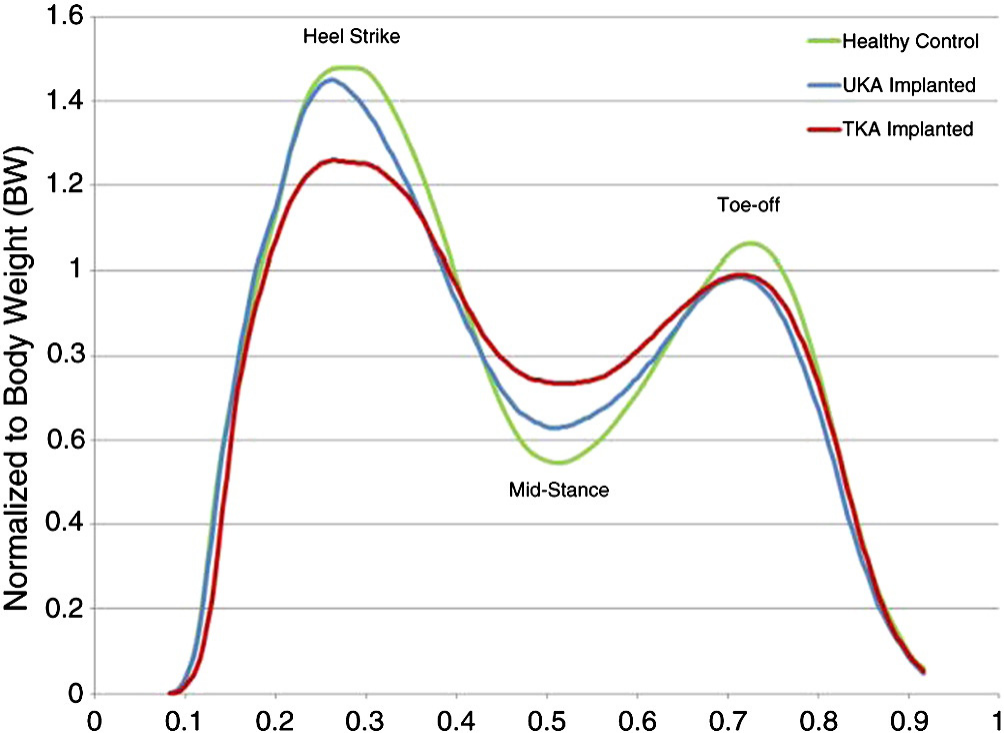
A graph of the mean Normalised Body Weight at Top Walking Speed, against the mean Normalised Stance Time for TKA (N = 21), UKA (N = 22), and the healthy control group (N = 14).

**Table 1:** Demographics of the 3 Study Groups, Unicompartmental Knee Arthroplasty (UKA N = 23), Total Knee Arthroplasty (TKA N = 23), and Normal Controls (N = 14).

Subject	UKA	TKR	Control
Gender M:F	10:13	9:14	6:8
Age (years)	65.9 ± 5.1	67.8 ± 7.7	60.0 ± 7.6
Oxford Score	44.1 ± 3.3	40. ± 8.1	NA
BMI	30.0 ± 4.3	29.4 ± 3.8	24.9 ± 3.6
Height (cm)	170.1 ± 7.8	168.5 ± 12.4	170.3 ± 11.5

**Table 2:** Results at Top Walking Speed (TWS).

Subject	UKA	TKA	Control
Speed (km/h)	7.0 ± 0.6	6.2 ± 0.8 a,b	7.4 ± 0.6
Hof Speed (H)	0.23 ± 0.02^a^	0.21 ± 0.02 a,b	0.25 ± 0.02
Cadence (step/min)	133 ± 16	131 ± 8^a^	139 ± 13
Heel Strike Force (BW)	1.52 ± 0.11	1.38 ± 0.18a,b	1.56 ± 0.13
Mid-Stance Force (BW)	0.58 ± 0.13	0.67 ± 0.13a,b	0.52 ± 0.11
Toe-Off Force (BW)	1.01 ± 0.13	1.01 ± 0.14	1.05 ± 0.09
Step Length (cm)	92 ± 9	87 ± 10 a	97 ± 8
Stride Length (cm)	189 ± 29	172 ± 20 a,b	190 ± 17
Impulse (BW/s)	0.45 ± 0.03	0.48 ± 0.03a,b	0.45 ± 0.05
Stance Time(s)	0.56 ± 0.03	0.60 ± 0.04a,b	0.56 ± 0.05
Gait Width (cm)	12.6 ± 2.7 a	12.6 ± 3.8	10.7 ± 2.4

The values are indicated as means ± standard deviation; BW = normalized to body weight; NIR = Normalized impulse ratio; Hof Speed (speed scaled by size as described by Hof) = dimensionless.

a Significant difference between implant versus control (*P* < 0.05).

b Significant difference between implant groups (*P* < 0.05).
