# Supplementary material for: Unicompartmental Knee Arthroplasty Enables Near Normal Gait at Higher Speeds, Unlike Total Knee Arthroplasty
Source: J Arthroplasty. 2013 Oct;28(9):176–8. doi: 10.1016/j.arth.2013.07.036 (PMC3809509; doi:10.1016/j.arth.2013.07.036)
Supplement: Supplementary file 4 — HP0008. [file mmc4.pdf]

CONFLICT OF INTEREST STATEMENT

The Journal of Arthroplasty

(Adopted from the American Academy of Orthopaedic Surgeons disclosure statement)

The following form **must be filled out completely and submitted by each author (example, 6 authors, 6 forms)**. **If no discloser is required, please write/type "none" at the end of each sentence.**

Manuscript Title

1. Royalties from a company or supplier (The following conflicts were disclosed) None

2. Speakers bureau/paid presentations for a company or supplier (The following conflicts were disclosed) None

3A. Paid employee for a company or supplier (The following conflicts were disclosed) None

3B. Paid consultant for a company or supplier (The following conflicts were disclosed) None

3C. Unpaid consultants for a company or supplier (The following conflicts were disclosed) None

4. Stock or stock options in a company or supplier (The following conflicts were disclosed) None

5. Research support from a company or supplier as a Principal Investigator (The following conflicts were disclosed) None

6. Other financial or material support from a company or supplier (The following conflicts were disclosed) None

7. Royalties, financial or material support from publishers (The following conflicts were disclosed) None

8. Medical/Orthopaedic publications editorial/governing board (The following conflicts were disclosed) None

9. Board member/committee appointments for a society (The following conflicts were disclosed) None

Each author must sign AND print or type his/her name, date and submit a separate form

In addition, one BLINDED Conflict of Interest form (no author names used) should be submitted per manuscript with all author disclosures.

Robin K Strachan	[signature]	11/02/2013
Author Name (Print or Type)	Author Signature	Date
