# Supplementary material for: Unicompartmental Knee Arthroplasty Enables Near Normal Gait at Higher Speeds, Unlike Total Knee Arthroplasty
Source: J Arthroplasty. 2013 Oct;28(9):176–8. doi: 10.1016/j.arth.2013.07.036 (PMC3809509; doi:10.1016/j.arth.2013.07.036)
Supplement: Supplementary file 6 — COI (2). [file mmc6.docx]

# CONFLICT OF INTEREST STATEMENT

***The Journal of Arthroplasty***

(Adopted from the American Academy of Orthopaedic Surgeons disclosure statement)

The following form **must be filled out completely and submitted by each author (example, 6 authors, 6 forms). If no discloser is required please write/type “none” at the end of each sentence.**

Predicting Femoral Head Diameter and Lesser Trochanter to Center of Femoral Head Distance: A Novel Method of Templating Hip Hemiarthroplasty

1. Royalties from a company or supplier (The following conflicts were disclosed) None

2. Speakers bureau/paid presentations for a company or supplier (The following conflicts were disclosed) None

3A. Paid employee for a company or supplier (The following conflicts were disclosed) None

3B. Paid consultant for a company or supplier (The following conflicts were disclosed) United HealthCare; Goldman Sachs

3C. Unpaid consultants for a company or supplier (The following conflicts were disclosed) None

4. Stock or stock options in a company or supplier (The following conflicts were disclosed) None

5. Research support from a company or supplier as a Principal Investigator (The following conflicts were disclosed)

None

6. Other financial or material support from a company or supplier (The following conflicts were disclosed) None

7. Royalties, financial or material support from publishers (The following conflicts were disclosed) None

8. Medical/Orthopaedic publications editorial/governing board (The following conflicts were disclosed)

Journal of Arthroplasty, Clinical Orthopaedics and Related Research

9. Board member/committee appointments for a society (The following conflicts were disclosed) None

**Each Author must Sign, Date and Submit a separate Form. Typed or Printed name must accompany signature.**

In addition, one BLINDED Conflict of Interest form (no author names used) should be submitted per manuscript with all author disclosures.


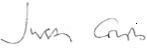
 05/08/2013

Author Signature Date
